# Supplementary material for: Motor control of Drosophila feeding behavior
Source: eLife. 2017 Feb 17;6:e19892. doi: 10.7554/eLife.19892 (PMC5315463; doi:10.7554/eLife.19892)
Supplement: Supplementary file 1. — Numbers are shown as flies displaying phenotype/total flies analyzed.Experimental flies (Gal4/UAS) were compared to control flies (w1118; Gal4/+; UAS/+) using a Wilcoxon signed-rank test.* GMR26A01, chaGal80>Chrimson flies were starved for 24 hr prior to testing. DOI: http://dx.doi.org/10.7554/eLife.19892.035 [file elife-19892-supp1.docx]

Schwarz et al. Supplementary Table 1

| Figure | Genotype | Gal4-line | UAS-line | | |
| --- | --- | --- | --- | --- | --- |
|  |  |  | TrpA1 | Chrimson | shibire^ts^ |
| Figure 5 | wildtype | GMR18B07 | 0/24 | 0/20 | 0/10 |
|  | Gal4 / + |  | 0/25 | 0/22 |  |
|  | Gal4, repo-Gal80 / + |  | 0/22 | 0/22 | 0/11 |
|  | UAS / + |  | 0/23 | 0/31 | 0/10 |
|  | Gal4 / UAS |  | 30/33  p<0.0001 | 43/45  p<0.0001 |  |
|  |  |  |  |  |  |
|  | Gal4, repo-Gal80 / UAS |  | 27/28  p<0.0001 | 55/55  p<0.0001 | 15/15  p<0.0001 |
|  |  |  |  |  |  |
|  |  |  |  |  |  |
| Figure 6 | wildtype | GMR26A01 | 0/24 | 0/20 | 0/10 |
|  | Gal4 / + |  | 0/22 | 0/19 | 0/12 |
|  | Gal4, cha-Gal80 / + |  | 0/21 | 0/22 |  |
|  | UAS / + |  | 0/23 | 0/31 | 0/10 |
|  | Gal4 / UAS |  | 38/53  p<0.0001 | 37/49  p<0.0001 | 24/25  p<0.0001 |
|  |  |  |  |  |  |
|  | Gal4, cha-Gal80 / UAS |  | 0/24 | 4/67*  p=0.125 | 0/12 |
|  |  |  |  |  |  |
|  |  |  |  |  |  |
| Figure 7 | wildtype | GMR81B12 | 0/24 | 0/20 | 0/10 |
|  | Gal4 / + |  | 0/26 | 0/23 | 0/10 |
|  | UAS / + |  | 0/23 | 0/31 | 0/10 |
|  | Gal4 / UAS |  | 46/47  p<0.0001 | 70/70  p<0.0001 | 15/16  p<0.0001 |
|  |  |  |  |  |  |
|  |  |  |  |  |  |
| Figure 8 | wildtype | GMR58H01 | 0/24 | 0/20 |  |
|  | Gal4 / + |  | 0/23 | 0/21 |  |
|  | UAS / + |  | 0/23 | 0/31 |  |
|  | Gal4 / UAS |  | 27/38  p<0.0001 | 45/49  p<0.0001 | 0/12 |
|  |  |  |  |  |  |
|  |  |  |  |  |  |
| Figure 8  -fig sup1 | wildtype | VT020958 | 0/24 | 0/20 |  |
|  | Gal4 / + |  | 0/24 | 0/24 |  |
|  | UAS / + |  | 0/23 | 0/31 |  |
|  | Gal4 / UAS |  | 33/43  p<0.0001 | 39/46  p<0.0001 | 0/12 |
|  |  |  |  |  |  |
